# Making sense of carbonic anhydrase function in zebrafish using antisense morpholinos

**DOI:** 10.1007/s00438-025-02303-0

**Published:** 2025-10-17

**Authors:** Ashok Aspatwar

**Affiliations:** Faculty of Medicine and Health Technology, Tampere University, 33520 Tampere, Finland

**Keywords:** Carbonic anhydrase, Morpholino, Zebrafish, Antisense oligonucleotides, Gene knockdown, Developmental biology, pH regulation, Pigmentation, Neurodevelopment

## Abstract

Understanding gene function in vertebrate development requires tools that allow precise and timely manipulation of gene expression. Zebrafish (*Danio rerio*), with its transparent embryos and rapid development, offers an ideal model to study vertebrate biology. This review explores how morpholino oligonucleotides (MOs), a widely used tool for transient gene knockdown, have been employed to investigate the roles of carbonic anhydrases (CAs) and carbonic anhydrase-related proteins (CARPs) in zebrafish. CAs are metalloenzymes, while CARPs are inactive isozymes that play critical roles in pH regulation, ion transport, CO₂ metabolism, and protein interactions influencing diverse biological functions. Many of the MO knockdown studies presented here have been extensively conducted in our laboratory over the past decade, revealing novel roles for CAs in neural development, reproduction, and swim bladder formation. These studies also confirm roles previously reported in humans, such as pigmentation, acid–base homeostasis, neural development, and motor coordination. We discuss technical aspects of MO design, delivery, and validation, and address common challenges such as off-target effects, transient gene silencing, and the necessity of rescue experiments. In addition, the review includes a comparative analysis of MOs versus CRISPR/Cas9-based genome editing, underscoring their respective advantages and limitations for functional genomics. In conclusion, this review provides not only a methodological guide but also biological insights into CA function in zebrafish, highlighting how antisense technology continues to inform vertebrate development and disease modeling. The lessons learned here may inform the study of other gene families and support translational research in carbonic anhydrase-related human disorders.

## Introduction

Understanding gene function during early vertebrate development requires model systems that are genetically tractable, physiologically relevant, and experimentally accessible. Among available models, the zebrafish (*Danio rerio*) stands out for its unique advantages in developmental biology and functional genomics. The zebrafish (*Danio rerio*) has become a widely used vertebrate model in developmental biology and genetics due to its genetic tractability, optical transparency during embryogenesis, rapid external development, and high fecundity (Aspatwar et al. 2022a; Howe et al. 2013). These features allow precise gene manipulation and real-time observation of gene function during early development. Moreover, its conserved physiology and high genetic homology with humans make it a powerful model for studying human disease genes. Importantly, zebrafish share considerable genetic homology with humans: approximately 69% of zebrafish genes have at least one human ortholog, and conversely, 71% of human genes have a zebrafish ortholog. Furthermore, 82% of human disease-causing genes are conserved in zebrafish (Howe et al. 2013).

Over the past two decades, antisense morpholino oligonucleotides (MOs) have become a fundamental tool for transient gene knockdown in zebrafish embryos (Nasevicius and Ekker 2000). This technique enables rapid assessment of gene function through loss-of-function phenotypes without the lengthy process of generating stable mutants. Despite increasing recognition of their limitations, including transient gene suppression, potential off-target toxicity, and activation of stress pathways, morpholinos continue to be widely used in zebrafish for early-stage functional analyses when designed and validated appropriately (Kok et al. 2015; Lai et al. 2019).

Carbonic anhydrases (CAs) comprise a large and evolutionarily conserved family of zinc metalloenzymes that catalyze the reversible hydration of carbon dioxide to bicarbonate and protons (Aspatwar et al. 2022b). Eight CA families (α, β, γ, δ, ζ, η, θ, and ι) have been identified across living organisms (Aspatwar et al. 2022b). Humans possess 13 enzymatically active CA isozymes and three carbonic anhydrase-related proteins (CARPs), which lack enzymatic activity but play putative regulatory roles, particularly in the nervous system (Aspatwar et al. 2010a, 2010b, 2013a, 2014). Active CA enzymes are critical for acid–base balance, ion transport, gas exchange, and diverse physiological processes in both humans and zebrafish (Aspatwar et al. 2021, 2022b, 2023). Zebrafish harbor orthologs of all 13 active CA enzymes and the three CARPs, as well as additional isoforms that may aid aquatic adaptation and acid–base homeostasis, making them an excellent model for studying the functional roles of human CAs and CARPs (Aspatwar et al. 2022a).

Notably, certain zebrafish CA isoforms, such as ca10a and ca10b, were named to reflect their homology with two distinct human genes, CA10 and CA11, respectively (Aspatwar et al. 2010a). Phylogenetic and sequence analyses conducted in our laboratory have demonstrated that these genes are not zebrafish-specific paralogs but represent evolutionarily conserved orthologs of separate human CARPs (Aspatwar et al. 2010a). As such, functional studies involving ca10a and ca10b in zebrafish offer distinct insights into the biological roles of CA10 and CA11 in human physiology, particularly in the nervous system (Aspatwar et al. 2015).

Our laboratory has pioneered the application of morpholino-mediated gene knockdown to systematically investigate CA and CARP functions in zebrafish, aiming to elucidate their relevance to human biology (Aspatwar et al. 2013b, 2015; Patrikainen et al. 2017). Since the early 2010s, we have targeted multiple CA isoforms with MOs, uncovering novel developmental roles and physiological phenotypes such as, motor coordination, swim bladder inflation, and embryonic viability, some of which were not fully predicted by mammalian studies (Aspatwar et al. 2022a; Kaya et al. 2011; Türkmen et al. 2009). These phenotypic differences may be attributed to the unique advantages of zebrafish as a model system, including transparent embryos, external development, and fine spatiotemporal control of gene knockdown. In some cases, early developmental processes or subtle phenotypes are more easily visualized or quantified in zebrafish than in mammalian models, enabling the detection of roles that may be masked or compensated for in mice. These findings underscore the biological and technical insights gained through MO-based approaches.

This review provides a comprehensive synthesis of morpholino technology applied to zebrafish CA research, detailing technical aspects of MO design, delivery, and validation alongside biological insights. It also discusses challenges inherent to morpholinos, such as off-target effects and transient knockdown, and places their use in the context of emerging genome editing technologies like CRISPR/Cas9 (Aspatwar et al. 2015; Raja et al. 2020). By contextualizing this approach within the broader landscape of vertebrate developmental genetics, the review aims to highlight the enduring utility of morpholinos and their relevance to both basic biology and translational research.

## Background of morpholinos

**Fig. 1:**
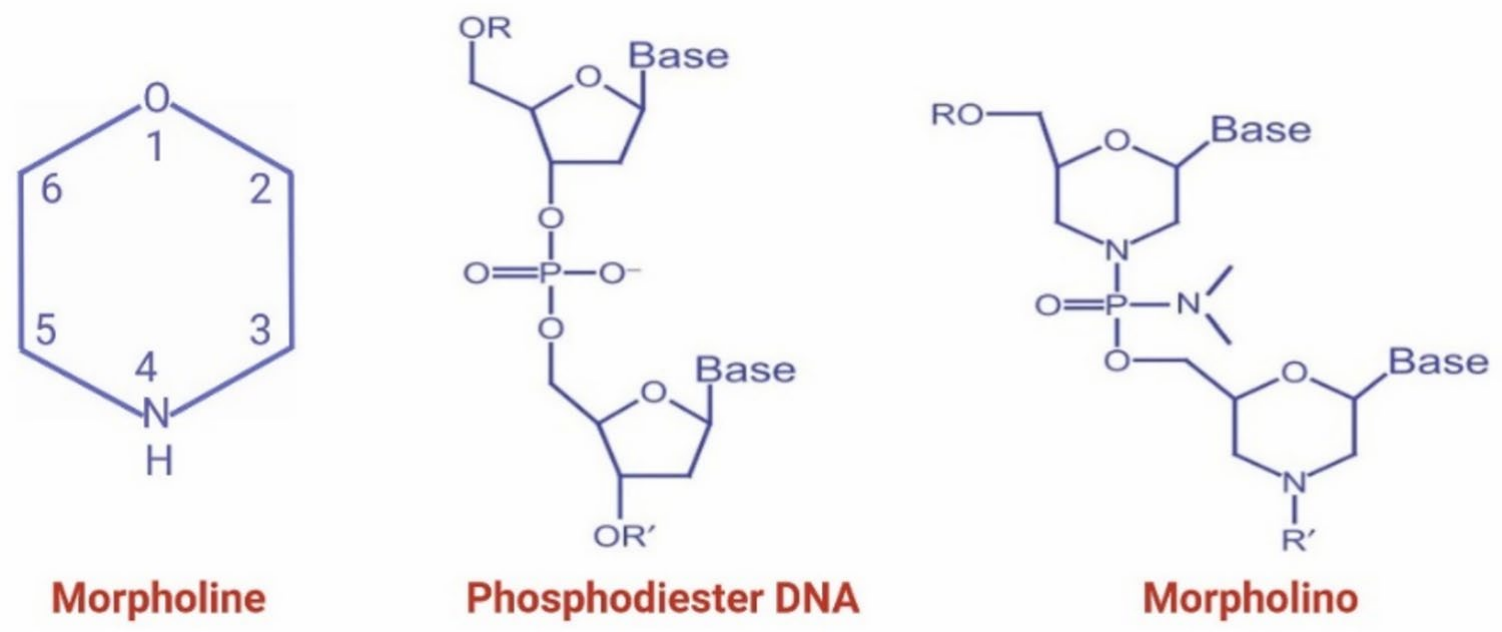
Chemical structure of morpholine (O(CH2CH2)2NH), the heterocyclic ring used to replace the ribose sugar in morpholino oligonucleotides, linked through phosphorodiamidate bonds

Antisense morpholino oligonucleotides (MOs) were developed as synthetic molecules designed to block gene expression by binding complementary RNA sequences. Morpholinos derive their name from the morpholine ring, a six-membered heterocyclic structure containing oxygen and nitrogen atoms, which replaces the ribose sugar found in natural nucleic acids. This morpholine backbone is linked by neutral phosphorodiamidate bonds, resulting in an uncharged, water-soluble molecule that can hybridize with high specificity and affinity to single-stranded RNA or DNA targets via Watson–Crick base pairing (Fig. 1). Unlike other antisense molecules such as siRNAs, morpholinos are resistant to endogenous nucleases, which imparts enhanced stability and minimizes degradation in biological systems (Summerton 2017).

Antisense morpholinos are designed to be complementary to specific mRNA or pre-mRNA sequences, thereby blocking translation or splicing of target genes. Although the initial concept of antisense morpholino chemistry emerged in the late 1980s, the first commercial availability of MOs suitable for gene knockdown in zebrafish embryos occurred in 1999 (Ekker 2000; Heasman 2002). Delivered into single-cell zebrafish embryos, morpholinos have revolutionized developmental biology by enabling rapid, transient gene knockdown without the need to generate stable mutant lines (Aspatwar et al. 2013b, 2015; Patrikainen et al. 2017; Ekker 2000; Heasman 2002).

Beyond zebrafish, morpholinos have been successfully applied in diverse organisms, including African clawed frogs (*Xenopus*), chickens (*Gallus gallus*), sea urchins (*Strongylocentrotus* spp.), sea squirts (*Ciona*), as well as in some bacterial, protist, plant, and fungal systems.

Morpholinos are commercially available from Gene Tools LLC providing an invaluable tool for gene function studies in model organisms with limited genetic resources (Corey and Abrams 2001). In this review, we focus on the application of morpholinos in zebrafish to suppress gene expression and elucidate gene function during embryonic development.

## Methodology and design of morpholinos for targeting carbonic anhydrase genes in zebrafish

Over the last two decades, morpholino oligonucleotides (MOs) have become indispensable tools for deciphering gene function during vertebrate development, particularly in zebrafish (Danio rerio). In the context of carbonic anhydrases (CAs), antisense MOs have enabled selective knockdown of gene expression, allowing detailed investigation of the physiological roles of various CA isoforms and carbonic anhydrase-related proteins (CARPs) in vivo.

Our laboratory has utilized MOs since 2010 to study the roles of several CA genes during zebrafish embryonic development. Studies on CA genes, including *ca8*, *ca10a*, *ca10b*, and *ca6*, have originated from our group (Aspatwar et al. 2022a). Other laboratories have contributed with MO-based knockdowns of *ca2* and c*a14* (Raja et al. 2020; Lin et al. 2008). Thus, this review not only synthesizes the field but also provides a firsthand methodological account.

Target Gene Identification and Sequence Verification The first step in MO-based knockdown is the precise identification of the target CA gene and verification of its transcript sequence.

- Zebrafish orthologs of CA genes were retrieved from genomic databases such as Ensembl and NCBI.
- Sequence verification was conducted by RT-PCR using primers designed to amplify full-length transcripts.
- PCR products from multiple individuals (n = 4–6) were sequenced to detect natural polymorphisms that might affect MO binding efficiency.
- This step is critical to ensure exact sequence matching, as even single mismatches can reduce MO efficacy.

Design of Translation-Blocking and Splice-Blocking Morpholinos For each CA gene, two types of MOs were designed:

- Translation-blocking MOs: Target the 5′ untranslated region (5′-UTR) and the start codon (AUG) to prevent ribosome assembly and inhibit translation initiation.
- Splice-blocking MOs: Bind to exon–intron or intron–exon splice junctions, leading to exon skipping or intron retention, thereby generating defective or truncated transcripts. Our studies typically employ both types to validate phenotypes and rule out off-target effects. MO design follows principles of:
- Perfect base-pair complementarity with the target sequence
- Minimal self-complementarity to avoid hairpins
- Low cross-reactivity with off-target transcripts, verified by BLAST analysis

Control Morpholinos and Validation Strategy To confirm specificity and control for off-target effects:

- Control MOs with scrambled sequences or five-base mismatches are used to establish baseline developmental outcomes.
- Co-injection of p53 MOs mitigates nonspecific apoptosis induced by some MOs.
- Rescue experiments typically involve co-injection of synthetic mRNA encoding the wild-type CA gene to reverse the morphant phenotype and confirm target specificity. However, successful rescue requires careful titration of mRNA, as overexpression may itself induce non-specific or toxic effects. In many cases, a lack of zebrafish-specific antibodies hinders validation of protein restoration, making it essential to test a range of mRNA concentrations to approximate endogenous expression levels. Thus, while rescue remains a key specificity control, its design and interpretation must be approached with caution.

Preparation and Verification of Morpholino Stock Solutions This protocol describes preparing morpholino stock solutions at 1 mM concentration suitable for microinjection into zebrafish embryos.

- Morpholino oligonucleotides (lyophilized powder) are supplied by Gene Tools.
- Use sterile, distilled, autoclaved water without DEPC.
- Prepare solutions in labeled 1.5 ml glass or polypropylene tubes.

Procedure:

1. Verify the quantity of morpholino on the vial label and accompanying documentation.
2. Using sterile technique, add an appropriate volume of sterile water to dissolve the MO at 1 mM (e.g., add 0.1 ml water to 100 nmol lyophilized MO).
3. Close vials tightly and mix thoroughly by gentle inversion.
4. Sterilize the solution by autoclaving using the liquid sterilization cycle.
5. Aliquot the stock solution into multiple microcentrifuge tubes, label each with concentration and MO identity, and store at room temperature for long-term use.

Microinjection of morpholinos into single-cell zebrafish embryos.

**Fig. 2:**
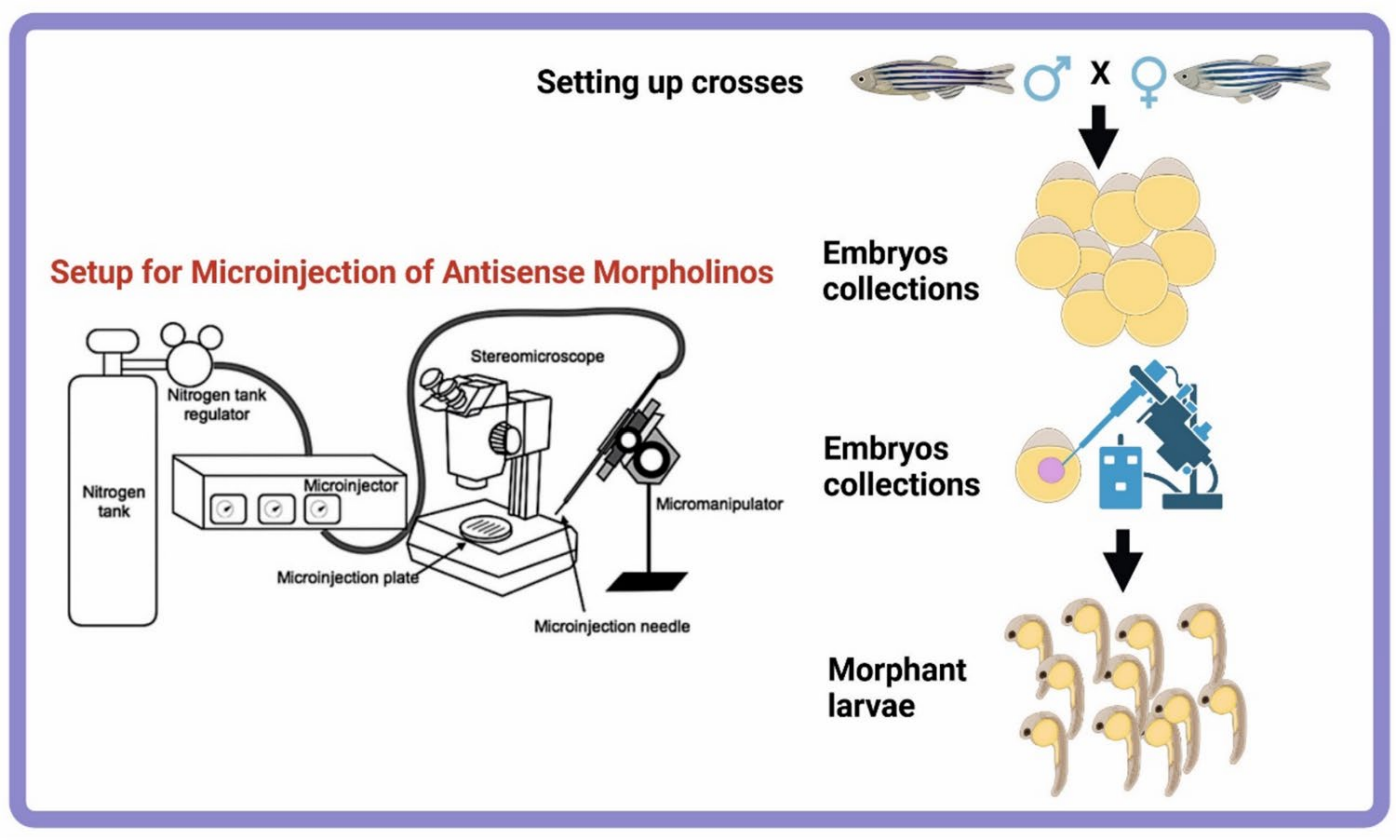
Method of gene manipulation using morpholinos in zebrafish embryos. This schematic illustrates the microinjection of morpholino oligonucleotides (MOs) into single-cell stage embryos and the subsequent phenotypic analysis to study gene function during zebrafish development

**Fig. 3:**
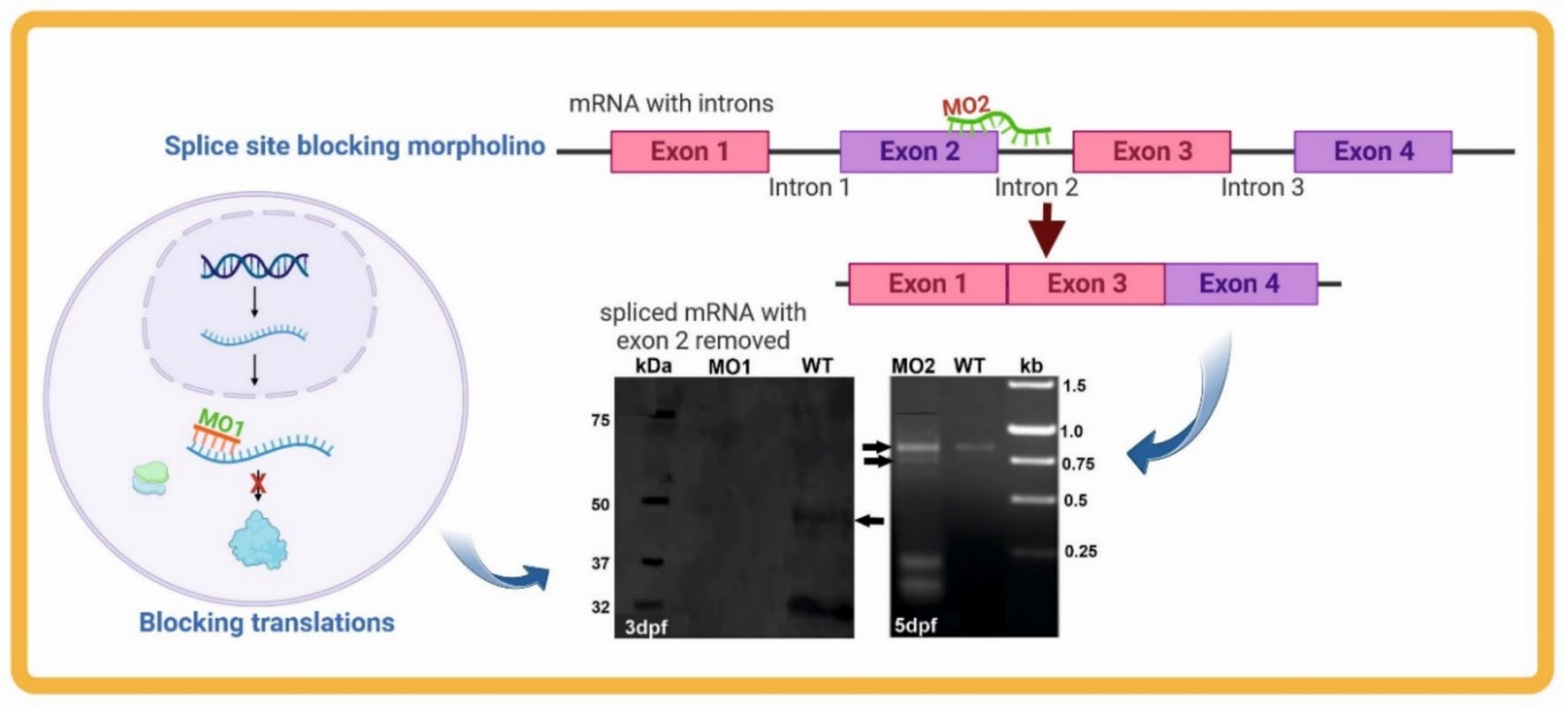
Molecular analysis of morphants to verify morpholino efficacy. The figure shows representative PCR and/or immunoblot assays used to confirm gene knockdown at the mRNA and protein levels in zebrafish embryos injected with MOs

1. Set up breeding pairs the day before injection to collect freshly fertilized embryos (Fig. 2).
2. Prepare microinjection setup including micromanipulator, microscope, injection needles, and injectate tracer dyes such as phenol red or rhodamine for visualization.
  Load glass capillary needles either by capillary action or using a pipette.
3. Inject approximately 1–2 nl of MO solution into the yolk or cytoplasm of single-cell stage embryos (Fig. 2).
4. Place injected embryos into Petri dishes containing standard zebrafish embryo medium (E3).
5. Incubate at 28.5 °C and monitor embryos for phenotypic changes indicative of gene knockdown.
6. Analyze gene knockdown efficacy by molecular methods such as RT-PCR or immunostaining (Fig. 3).

Stepwise Protocol for Designing Morpholino Knockdown Experiments in Zebrafish.

1. Gene selection: Identify the target zebrafish gene from genomic databases.
2. Expression verification: Design primers to amplify the full-length transcript and perform PCR.
3. Sequence confirmation: Sequence PCR products from 4–6 fish individuals to identify polymorphisms.
4. MO type selection: Use two MOs per gene — one translation-blocking and one splice-blocking.
5. Target region selection:
  o For translation blockers, use the mRNA 5′-UTR plus the first 25 coding bases.
  o For splice blockers, select pre-mRNA splice junctions (intron–exon or exon–intron) that, when blocked, cause defective transcripts.
6. Order control MOs: Obtain random sequence controls or five-base mismatch controls from Gene Tools.
7. Splice-block target choice: Choose splice junctions where MO binding causes frameshifts and activates nonsense-mediated decay, leading to mRNA degradation.
8. Design optimized MOs: Generate the inverse complement of the target RNA sequence, following targeting rules (detailed in commentary).
9. BLAST homology search: Confirm that the MO target sequence does not significantly match other mRNAs to avoid off-target effects. If homology is found, select alternate targets.

## Functional roles of carbonic anhydrase isoforms in zebrafish development

**Fig. 4:**
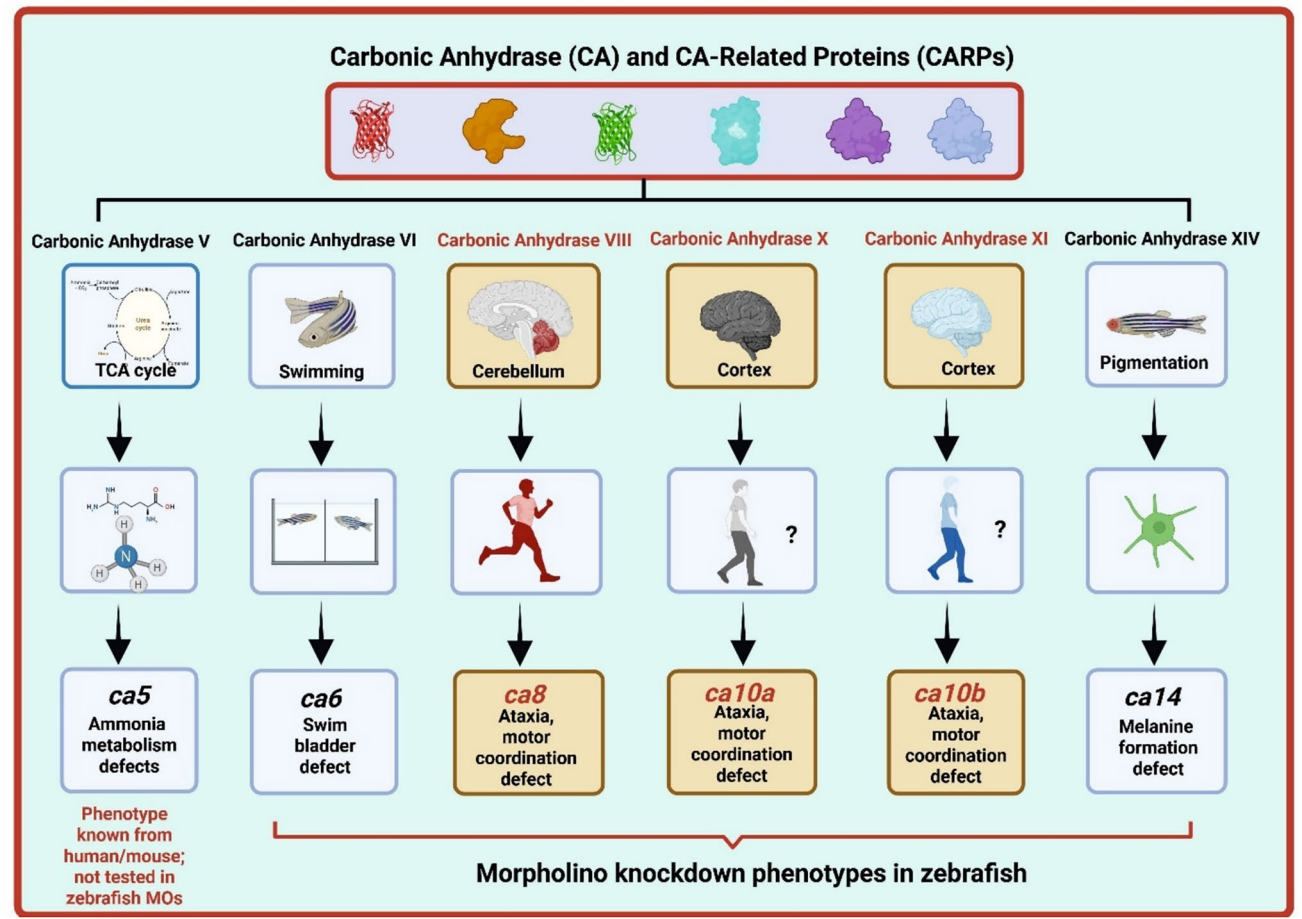
Functional roles and zebrafish knockdown phenotypes of selected carbonic anhydrase (CA) and CA-related protein (CARP) isoforms. This schematic summarizes the biological functions and morpholino-induced phenotypes of key CA and CARP isoforms in zebrafish. Catalytically active CAs (*ca5*, *ca6*, *ca14*) contribute to ammonia metabolism, swim bladder inflation, and pigmentation, while CARPs (*ca8*, *ca10a*, *ca10b*), inactive but functionally important, are associated with motor coordination and brain development. Morpholino knockdown phenotypes are shown in the lower panels. *ca5* function is based on human and mouse studies but has not been tested using morpholinos in zebrafish. Question marks (?) indicate areas of incomplete functional understanding. Isoforms with brown background represent CA-related proteins. This model integrates evidence from zebrafish, mammalian mutants, and human disease studies (Aspatwar et al. 2022a; Aspatwar et al. 2015) (These citations for Figure 4 are correct)

Carbonic anhydrases (CAs) in zebrafish perform diverse and isoform-specific functions critical for development, ion regulation, and organogenesis. Isoforms such as ca2a are localized to H⁺-ATPase-rich (HR) cells and play essential roles in chloride uptake and systemic acid–base homeostasis (Table 1) (Aspatwar et al. 2022a; Lin et al. 2008). ca5, a mitochondrial isoform, is expressed in the lens and developing pancreas, where it contributes to acid–base regulation and medial fin development. **ca6**, found in skin, heart, gills, and the swim bladder, is essential for swim bladder development and may also participate in immune defense (Aspatwar et al. 2022a; Postel and Sonnenberg 2012). ca8, though catalytically inactive, is highly expressed in the central nervous system, especially cerebellar Purkinje cells and plays a central role in motor coordination, particularly through its role in Purkinje cell function (Aspatwar et al. 2010b; Aspatwar et al. 2013b). Similarly, ca10a and ca10b, both CA-related proteins (CARPs), are expressed in neural tissues and the swim bladder; their suppression results in defective motor behavior and embryonic lethality, highlighting their non-catalytic but vital roles in neurodevelopment (Aspatwar et al. 2015). **ca9**, while not yet studied through knockdown in zebrafish, shows strong expression in hypoxia-sensitive tissues such as the eye, liver, and brain, suggesting a conserved role in oxygen sensing (Esbaugh et al. 2009). ca14 is implicated in melanocyte maturation and is expressed in the retinal pigmented epithelium and skeletal muscle, with knockdown resulting in pigmentation defects (Raja et al. 2020). Finally, zebrafish ca15a (also referred to as ca4c in zebrafish) and ca15b are expressed in HR cells and gills, contributing to sodium uptake and pH balance; morpholino knockdown causes pericardial edema and tail abnormalities (Table 1) (Ito et al. 2013; Tarbashevich et al. 2015). Together, these findings underscore the physiological importance of CA isoforms in zebrafish development, particularly in ion regulation, acid–base balance, pigmentation, and neuromuscular coordination, and establish zebrafish as a valuable model for dissecting both enzymatic and non-enzymatic CA functions in vivo (Aspatwar et al. 2022a) (Fig. 4).

**Table 1:** Summary of Morpholino-Mediated Knockdown Studies of Carbonic Anhydrases (CAs) and CA-Related Proteins (CARPs) in Zebrafish

Gene	MO type	Observed phenotype	Larval stage	Function affected region	Validation method(s)	References
*ca2a / ca2b*	Translation-blocking MO	Pericardial edema, craniofacial abnormalities, reduced pigmentation	24–72 hpf	Craniofacial cartilage, pigment cells	Rescue with human CA2 mRNA	Aspatwar et al. (2022a); Lin et al. (2008); Shiao et al. (2005)
*ca6*	Translation-blocking MO	Swim bladder deflation, buoyancy defects	4 dpf	Swim bladder, coordination	qRT-PCR, behavioral assessment	Patrikainen et al. (2017)
*ca8*	Translation-blocking and splice blocking MOs	Motor coordination defects	5dpf	Ataxia, cerebellum	qRT-PCR, behavioral assessment	Aspatwar et al. (2013b)
*ca10a*	Translation-blocking MO	Curved body, pericardial edema, brain apoptosis	24–72 hpf	CNS, head	TUNEL assay, CRISPR validation	Aspatwar et al. (2015)
*ca10b*	Translation-blocking MO	Eye and head defects, movement abnormalities	24–72 hpf	Brain, motor system	TUNEL assay, CRISPR validation	Aspatwar et al. (2015)
*ca14*	Splice-blocking MO	Pigmentation defects, delayed melanocyte differentiation	48–96 hpf	Melanocytes	CRISPR validation, H3K27ac levels	Raja et al. (2020)
*ca15a*	Translation-blocking MOs	Skin, gills, H-ATPase-rich (HR) cells	–	Na^+^ uptake, acid base regulation mechanism	–	Lin et al. (2008; Ito et al. (2013)
*ca15b*	^a^Knockdown	–	–	Disrupted primordial germ cell migration	–	Tarbashevich et al. (2015)

^a^Two non-overlapping morpholinos were used in the referenced study, but the exact MO type (translation- or splice-blocking) was not specified

## Advantages, limitations, and validation of morpholino knockdown in zebrafish

**Fig. 5:**
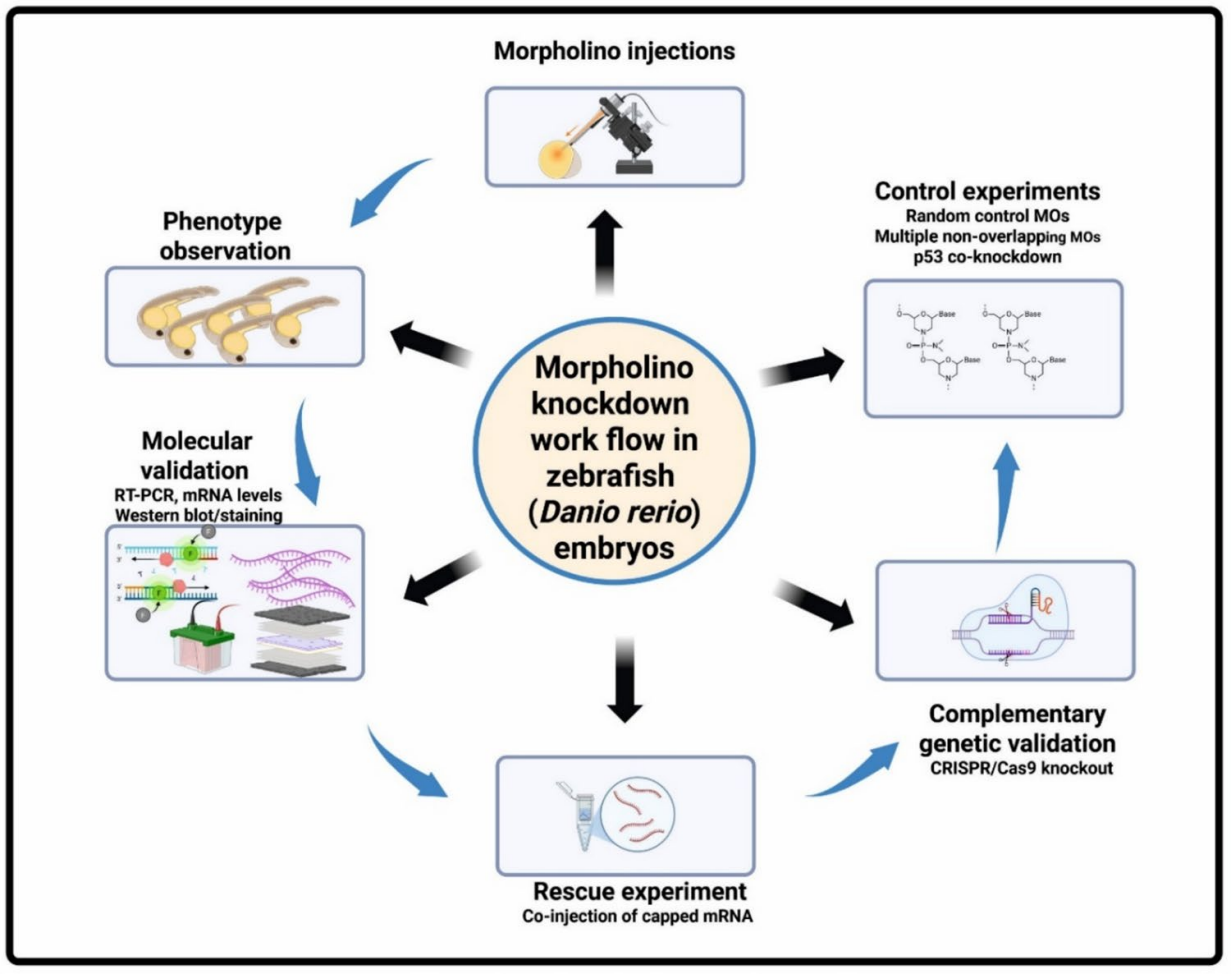
Workflow for Morpholino Knockdown in Zebrafish Embryos. Schematic representation of the standard experimental workflow used to assess gene function via morpholino (MO) antisense oligonucleotide injection in Danio rerio embryos. The process begins with targeted MO injection, followed by phenotypic observation of developing embryos. Molecular validation techniques such as RT-PCR and Western blotting confirm knockdown efficiency. Rescue experiments,co-injecting capped mRNA—help establish specificity. Critical control experiments include use of random control MOs, non-overlapping MOs, and p53 co-knockdown. Complementary genetic validation using CRISPR/Cas9 knockout provides further support for gene-specific effects. This integrative approach enhances the reliability of functional interpretations in zebrafish developmental studies

Morpholino oligonucleotides (MOs) have become a widely adopted and effective tool for transiently suppressing gene expression in zebrafish embryos, including for functional studies of carbonic anhydrase (CA) isoforms. Their ability to provide precise temporal control over gene knockdown during early developmental stages makes MOs particularly suited for investigating dynamic gene functions in vivo (Aspatwar et al. 2022a). Figure 5 presents the workflow of Morpholino Knockdown in Zebrafish Embryos.

A key advantage of MOs is their high specificity and remarkable stability. When carefully designed and validated, MOs can achieve reasonable target specificity; however, off-target effects and toxicity have been widely reported and must be addressed through rigorous controls. The chemically modified phosphorodiamidate backbone confers resistance to endogenous enzymatic degradation and ensures strong, sequence-specific binding to target RNA. This enables efficient inhibition of mRNA translation or pre-mRNA splicing (Aspatwar et al. 2015). Such properties are critical for studying developmental genes like CAs, where early expression patterns and resultant phenotypes offer valuable functional insights.

However, MOs are not without limitations. Off-target effects, including unintended activation of the p53-mediated apoptosis pathway, can confound phenotypic interpretation (Robu et al. 2007; Gerety and Wilkinson 2011). To mitigate these issues, rigorous experimental design is essential. This includes using multiple non-overlapping MOs targeting the same gene, implementing appropriate negative controls, and employing p53 co-knockdown strategies. Furthermore, the transient nature of morpholino knockdown, due to dilution as embryonic cells divide, restricts phenotypic assessment largely to early developmental stages, Table 2 shows advantages and limitations of MO knockdown studies.

**Table 2:** Advantages and limitations of morpholino knockdown in zebrafish

Advantages	Limitations
High specificity due to sequence complementarity	Possible off-target effects, including p53 activation
Chemically stable; resistant to enzymatic degradation	Transient knockdown; dilution during embryonic divisions
Effective inhibition of mRNA translation or splicing	Requires careful dose optimization
Rapid and relatively easy to administer by microinjection	Phenotypes limited mostly to early development stages
Widely available and well-characterized tool	Potential toxicity at high concentrations
Compatible with many model organisms	Requires rigorous controls and validation

Validation of morpholino-induced phenotypes is paramount to ensure specificity and biological relevance. In our CA studies, we confirmed knockdown efficiency and specificity using complementary techniques such as RT-PCR and Western blotting, as well as rescue experiments via co-injection of MO-resistant mRNA constructs (Aspatwar et al. 2022a; Aspatwar et al. 2015; Aspatwar et al. 2013b). Additionally, CRISPR/Cas9 gene editing has emerged as an invaluable orthogonal approach to corroborate morpholino results, helping to exclude off-target or transient effects. In summary, while morpholino oligonucleotides remain a powerful and rapid method for functional gene analysis in zebrafish, their inherent limitations highlight the necessity for meticulous validation and integration of complementary genetic approaches to draw robust conclusions.

## Comparison of morpholino knockdown and CRISPR/Cas9 gene editing in studying carbonic anhydrases in zebrafish

Morpholino oligonucleotides (MOs) are uncharged, highly specific antisense molecules that bind target RNAs with minimal toxicity and off-target interactions in cells. Their stability and resistance to enzymatic degradation make them effective tools for transient gene knockdown. Splice-blocking MOs allow exon-specific manipulation, enabling study of gene splice variants. However, MOs can induce off-target effects, including activation of Tp53-mediated apoptosis, necessitating careful experimental design with multiple MOs to replicate phenotypes. Additionally, MO effects are transient and diminish as embryonic cells proliferate, limiting their use to early developmental stages. Therefore, MO-induced phenotypes often require validation with complementary gene editing methods such as CRISPR/Cas9.

Morpholino knockdown and CRISPR/Cas9 gene editing are complementary approaches widely used for functional genomic studies in zebrafish, each with distinct strengths and limitations. Morpholinos provide rapid, transient suppression of gene expression during early embryogenesis, enabling quick functional screening and identification of gene roles during critical developmental windows. CRISPR/Cas9 enables stable, heritable gene disruption, allowing comprehensive analysis of gene function throughout development and adulthood.

Our investigations into carbonic anhydrase-related proteins (CARPs), including *ca10a* and *ca10b*, illustrate this synergy (Aspatwar et al. 2015). Initial MO knockdown revealed essential roles for these CARPs in neurodevelopment, manifesting as developmental delays, brain apoptosis, body axis curvature, pericardial edema, and impaired locomotion (Aspatwar et al. 2015, 2013b). To confirm specificity and address MO limitations, we generated CRISPR/Cas9 knockout mutants. These mutants recapitulated key morphant phenotypes, validating the MO results and underscoring the genes’ vital functions. Notably, both knockdown and knockout of *ca10a* and *ca10b* resulted in embryonic lethality by 3–5 days post fertilization, demonstrating their essentiality (Aspatwar et al. 2015). Similarly, studies on *ca14* using both methods enabled longitudinal phenotypic analyses beyond early embryogenesis, overcoming the temporal constraints of MO dilution during cell divisions (Raja et al. 2020).

In conclusion, morpholinos are well-suited for rapid, early-stage functional assessments and high-throughput genetic screens, but they require rigorous controls—such as the use of multiple independent MOs and rescue experiments—to confirm specificity. In contrast, CRISPR/Cas9 enables permanent gene disruption for detailed phenotypic analysis across all stages of development and into adulthood, although it involves longer generation times and may be subject to genetic compensation. An important consideration when comparing these approaches is that CRISPR/Cas9-induced mutations, especially those resulting in premature termination codons (PTCs), can activate transcriptional adaptation, leading to the upregulation of related genes that may mask expected phenotypes (Rossi et al. 2015; El-Brolosy et al. 2019; Ma et al. 2019). This compensatory mechanism is generally not triggered by morpholino-mediated knockdown, which does not alter the genome. As a result, phenotypic discrepancies between morphants and CRISPR mutants may not necessarily reflect MO off-target effects, but rather compensatory gene expression uniquely associated with CRISPR-induced mutations. The combined use of both technologies provides a robust and complementary framework for dissecting the developmental and physiological roles of carbonic anhydrases and their related proteins in zebrafish (Table 3).

**Table 3:** Comparison of morpholino knockdown and CRISPR-cas9 gene editing in zebrafish CA studies

Gene	Tool used	Phenotype	MO–CRISPR phenotype	Validation	References
*ca10a*	MOs and CRISPR	Curved body, CNS apoptosis, Movement disorder	High	CRISPR confirmed MO results	Aspatwar et al. (2015)
*ca10b*	MOs and CRISPR	Brain/head defects, swim defects	High	MO findings supported by CRISPR KO	Aspatwar et al. (2015)
*ca14*	MOs and CRISPR	Hypopigmented, immature melanocytes	Strong	pH-regulated epigenetic mechanism validated	Raja et al. (2020)

## Summary of morpholino and CRISPR studies on carbonic anhydrases in zebrafish

To provide a comprehensive overview of functional studies targeting carbonic anhydrase (CA) isoforms in zebrafish, we compiled two summary tables. Table 1 presents morpholino (MO)-based knockdown studies, highlighting the targeted CA isoforms, observed phenotypes, and key references, demonstrating the utility and insights gained from transient gene suppression. Table 2 summarizes CRISPR/Cas9 gene-editing studies, detailing the effects of permanent CA gene disruption. This allows direct comparison of phenotypic outcomes and validation of MO results.

Together, these tables illustrate the effectiveness of morpholinos in uncovering CA functions during early development, while also emphasizing the complementary role of CRISPR/Cas9 in confirming these findings and enabling longer-term functional analyses. The integration of both approaches provides a robust framework for elucidating the roles of CA isoforms in zebrafish biology.

## Refining functional studies through combined methods

While both morpholino and CRISPR/Cas9 technologies have been instrumental in studying carbonic anhydrase function in zebrafish, neither technique alone can provide a complete picture. Morpholinos enable early-stage knockdown by blocking mRNA translation or splicing, but concerns such as transient effects and off-target phenotypes persist. Conversely, CRISPR/Cas9 offers precise genome editing, yet its efficiency can be limited by chromatin accessibility, as highlighted in zebrafish by Uusi-Mäkelä et al. (2018). Combining the two methods has proven particularly effective. Our own studies on *ca10* and *ca10b* demonstrated that CRISPR-mediated knockout confirmed and extended the phenotypes observed in morphants, reinforcing the biological validity of our findings. Similarly, Raja et al. (2020) employed both MO and CRISPR approaches in their investigation of *ca14*, linking carbonic anhydrase function to melanocyte differentiation through pH-regulated histone acetylation. These studies underscore the strength of an integrated strategy, where morpholino-based observations are validated and refined by stable CRISPR knockouts, ultimately enabling more reliable and nuanced interpretations of gene function in zebrafish.

The type of morpholino used, translation-blocking vs. splice-blocking, can significantly influence the observed phenotype due to differential engagement of genetic compensation mechanisms. Splice-blocking MOs often introduce premature termination codons (PTCs), leading to nonsense-mediated mRNA decay (NMD), which can in turn trigger transcriptional adaptation and upregulation of gene paralogs or related pathways (El-Brolosy et al. 2019; Ma et al. 2019; Falcucci et al. 2025). In contrast, translation-blocking MOs typically do not activate such responses, as the mRNA remains intact. Although this phenomenon has not been specifically characterized for CA or CARP morphants in zebrafish, our ongoing work aims to dissect potential compensation effects by comparing phenotypes from both MO types and corresponding CRISPR/Cas9 mutants. Recent studies have highlighted that morpholinos may induce off-target phenotypes, including p53 activation, unrelated to their intended gene target (Kok et al. 2015). While p53 co-injection is a common control to suppress apoptosis-related artifacts, it is now understood that morpholinos can also activate broader stress responses, including innate immune and interferon pathways (Lai et al. 2019). These immune signatures can introduce additional confounding variables in phenotype interpretation and underscore the need for careful experimental design and validation using orthogonal methods. As such, proper controls, including mismatch MOs, rescue experiments, and alternative validation strategies (e.g., CRISPR), are essential to confirm the specificity of observed phenotypes.

The combined application of morpholino-mediated knockdown and CRISPR-based gene editing has emerged as a complementary strategy for uncovering the diverse roles of carbonic anhydrases in zebrafish. This dual approach not only enhances the reliability of functional interpretations but also bridges early developmental insights with long-term phenotypic validation. Looking ahead, the integration of emerging tools, such as single-cell transcriptomics, high-resolution live imaging, and multiplexed gene perturbation, promises to further refine our understanding of CA isoform-specific functions in vertebrate biology and disease models.

An emerging alternative to morpholinos for rapid gene function assessment is the use of CRISPants, F0 embryos injected with pre-assembled CRISPR/Cas9 components. CRISPants offer many of the advantages of morpholinos, including early phenotypic analysis, without the toxicity and off-target effects commonly associated with MOs. When efficient guide RNAs are used, CRISPants can achieve high knockout rates that result in observable phenotypes during early development. While mosaicism remains a challenge, recent advances in multiplexed guide RNA design and high-fidelity Cas9 variants have improved mutational consistency. CRISPants have been particularly useful in high-throughput screens and proof-of-principle functional studies, and their use is rapidly increasing in zebrafish research.

It is important to note, however, that phenotypes observed in MO-based studies are not always replicated in CRISPR mutants. Discrepancies can arise from MO-induced toxicity, genetic compensation in stable knockouts, or differences in timing and dosage of gene disruption (Kok et al. 2015; Rossi et al. 2015). These differences highlight the need for cautious interpretation and ideally, the use of complementary approaches to validate gene function.

## Summary and conclusions

The combined use of morpholino oligonucleotides (MOs) and CRISPR/Cas9 gene editing has significantly advanced our understanding of carbonic anhydrases (CAs) and carbonic anhydrase-related proteins (CARPs) in zebrafish development and physiology. MOs provide a rapid and effective means to transiently suppress gene expression during early embryogenesis, enabling the identification of critical developmental roles for various CA isoforms, such as in pigmentation, acid–base regulation, and motor coordination. However, due to their transient nature and potential off-target effects, MOs alone may not fully capture the gene’s function throughout development.

CRISPR/Cas9 technology complements this by offering precise, stable, and heritable gene disruption, allowing detailed functional analyses across all life stages and overcoming limitations associated with morpholino knockdown. MO knockdown and CRISPR/Cas9 comparative studies of genes such as ca10a, ca10b, and ca14 demonstrate that phenotypes observed in morphants are largely recapitulated in CRISPR mutants, thereby validating the specificity of MO-based approaches and enhancing confidence in functional interpretations.

This synergy between MOs and CRISPR/Cas9 has been particularly valuable for dissecting the complex and sometimes unexpected roles of carbonic anhydrases in zebrafish biology. Employing both MOs and CRISPR/Cas9 tools provides a robust framework for gene function studies, reducing false positives and negatives and enabling comprehensive exploration of gene families with multiple CA isoforms.

Despite these advances, some challenges remain. Off-target effects and transient knockdown complicate morpholino-based studies, while CRISPR/Cas9 approaches can produce mosaicism and genetic compensation. Continued improvements in targeting specificity and validation methods are essential. Emerging genome editing technologies, such as base editing and prime editing, offer promising avenues for precise, efficient, and less disruptive gene modification.

In conclusion, integrating transient (MO) and stable (CRISPR) gene perturbation methods strengthens the rigor and depth of zebrafish carbonic anhydrase research, setting a methodological precedent for future vertebrate gene function studies.

## Data Availability

Not applicable.
